# Supplementary material for: Groundwater discharge as a driver of methane emissions from Arctic lakes
Source: Nat Commun. 2022 Jun 27;13:3667. doi: 10.1038/s41467-022-31219-1 (PMC9237097; doi:10.1038/s41467-022-31219-1)
Supplement: Supplementary file 1 — Supplementary Information [file 41467_2022_31219_MOESM1_ESM.pdf]

Supplementary Information for

Groundwater discharge as a driver of methane emissions from Arctic lakes

Carolina Olid[1,2,3*], Valentí Rodellas[4], Gerard Rocher-Ros[1], Jordi Garcia-Orellana[4,5], Marc Diego-Feliu[4,5,6,7], Aaron Alorda-Kleinglass[4], David Bastviken[8], Jan Karlsson

[1]Climate Impacts Research Centre, Department of Ecology and Environmental Science, 90187 Umeå, Sweden.

[2]Department of Forest Ecology and Management, Swedish University of Agricultural Science, 90183 Umeå, Sweden.

[3]UB-Geomodels Research Institute, Departament de Dinàmica de la Terra i l'Oceà, Facultat de Ciències de la Terra, Universitat de Barcelona, 08028 Barcelona, Spain.

[4]Institut de Ciència i Tecnologia Ambientals, Universitat Autònoma de Barcelona, 08193 Bellaterra, Spain.

[5]Departament de Física, Universitat Autònoma de Barcelona, 08193 Bellaterra, Spain.

[6]Department of Civil and Environmental Engineering, Universitat Politècnica de Catalunya, 08034 Barcelona, Spain

[7]Associated Unit: Hydrogeology Group, UPC-CSIC, 08034 Barcelona, Spain

[8]Department of Thematic Studies – Environmental Change, Linköping University, 58183 Linköping, Sweden.

*Corresponding author

Email: carolina.olid@ub.edu

Supplementary Methods

S.1 Gas transfer velocity (*k*)

The gas transfer velocity (*k*) was calculated by using the empirical equation:

$$k = k_{600}(S_c/600)^{-n} \quad \text{(S1)}$$

where S_c is the Schmidt number for the corresponding gas at the measured water temperature[1] (S_c is divided by 600 to normalize to CO_2 at 20ºC), and *n* was given the value of 0.5 for wind speed > 3.6 m s^{-1} or 2/3 for wind speed < 3.6 m s^{-1}.

Estimates of means and uncertainties in k_{600} were calculated using the model developed by Klaus and Vachon[2] as follows:

$$k_{600} = [0.328 \cdot \log_{10}(A) + 1.581] \cdot U_{10} - 0.066 \cdot \text{logit}(SIN) + 1.266 \quad \text{(S2)}$$

where U_{10} [m s^{-1}] is the wind speed at a height of 10 m, *A* is the lake area [km^2], and *SIN* is the scale of spatial integration, which corresponds to the whole-lake in mass balance approaches as the one used here (*SIN* = 1). Here, U_{10} was calculated from the measured wind speed (U_z) following[3]:

$$U_{10} = U_z\left\{1 + \left[(C_{d10})^{1/2}/\kappa\right] ln(10/z)\right\} \quad \text{(S3)}$$

where C_{d10} is the drag coefficient at 10 m height (0.0013), κ is von Karman's constant (0.41), and z is the height of the sensor.

S.2 ^{222}Rn mass balance

Groundwater discharge rates were estimated by establishing a ^{222}Rn mass balance that accounts for all possible inputs and outputs of ^{222}Rn[4,5] (Supplementary Figure 5). This approach assumes that groundwater discharge into the study lakes is in steady state for short periods (days to weeks), and the water column is well mixed. Our surveys showed that the study lakes are generally uniform with respect to ^{222}Rn distribution. Sites of anomalous ^{222}Rn concentrations were rare and did not follow any clear pattern to explain the high measured ^{222}Rn concentrations. The values used to estimate all terms in the ^{222}Rn mass balance are summarized in Supplementary Tables 1 and 2. Sources of uncertainty for each term are described in Supplementary Table 3.

<u>Sinks of ^{222}Rn</u>

- *Atmospheric ^{222}Rn evasion*

The loss of ^{222}Rn to the atmosphere (f_{atm}) was determined using Equation 2 provided in the main text and a wind-based model developed by Klaus and Vachon[2] for k parametrization. Here, we assumed that atmospheric ^{222}Rn concentration was negligible compared to lake ^{222}Rn concentration ($C_{Rn,air}$ = 0). Integrating wind-speed data for the ^{222}Rn residence time in the lakes (~3 days prior to sampling) and weighting the influence of degassing on ^{222}Rn concentrations attending the proximity to the sampling time[6], k_{Rn} estimates ranged from 0.43 to 1.3 m d^{-1}. Resulting losses of ^{222}Rn to the atmosphere ranged from 9 to 170 Bq m^{-2} d^{-1}, accounting for 23 – 69% of the total ^{222}Rn losses.

- *^{222}Rn decay*

Radon losses due to radioactive decay (F_{decay}) are the product of the ^{222}Rn inventory [Bq m^{-2}] in the lake by the ^{222}Rn decay constant [d^{-1}]. The inventory of ^{222}Rn was obtained by multiplying the lake ^{222}Rn concentration [Bq m^{-3}] by the mean depth of the lake [m]. ^{222}Rn losses due to decay ranged from 7 to 124 Bq m^{-2} d^{-1}. Radioactive decay accounted for 30 – 76% of the total ^{222}Rn losses.

- *^{222}Rn export through outlet streams*

Direct export of ^{222}Rn through the outlet streams (F_{outlet}) was estimated by multiplying the estimated outlet discharge (Q_{outlet}, estimated using a magnetic flow meter or salt slug injections) [m^3 d^{-1}] by the ^{222}Rn concentration in the outlet [Bq m^{-3}]. The flux of ^{222}Rn lost through the outlet streams was then estimated by dividing this export by the lake area. Losses of ^{222}Rn through the outlet streams ranged from 0.24 to 71 Bq m^{-2} d^{-1}. The ^{222}Rn loss through the outlet streams accounted for 0.19 – 31% of the total ^{222}Rn losses.

<u>Sources of ^{222}Rn</u>

- *Production of ^{222}Rn via decay of dissolved ^{226}Ra*

Inputs of ^{222}Rn via decay of dissolved ^{226}Ra (F_{Ra}) were estimated from the dissolved ^{226}Ra inventory [Bq m^{-2}] obtained by multiplying the measured dissolved ^{226}Ra concentration in the lake [Bq m^{-3}] by the mean lake depth [m]. Dissolved ^{226}Ra was detected only in two lakes (detection limit of 6 Bq kg^{-1}), with an average concentration of 16.0 ± 7.4 Bq kg^{-1}. The flux of ^{222}Rn by ^{226}Ra decay was obtained by multiplying the average dissolved ^{226}Ra inventory by the decay constant of ^{222}Rn [d^{-1}], leading to 0.67 ± 0.15 Bq m^{-2} d^{-1}. Because ^{222}Rn production via ^{226}Ra decay only represents 0.23 – 3.1% of the total ^{222}Rn inputs, we considered this term

negligible in the ^{222}Rn mass balance for those lakes where ^{226}Ra in lake water was not quantified.

- *Diffusion of ^{222}Rn from sediments*

^{222}Rn produced in the sediments can be transported to the overlying water through diffusion (F_{diff}) or advection (named here as groundwater inflow, Q_{gw}). Constraining ^{222}Rn inputs due to groundwater advection requires thus subtracting inputs via diffusion. The diffusive ^{222}Rn flux from underlying sediments was assessed using incubated sediments in the lab[7]. Briefly, ca. 150-200 g of wet lake sediments were hermetically sealed into 500 mL PET bottles with Ra-free lake water prefiltered with MnO_2 fibers. The bottle was connected in a close loop to the RAD7 radon-in-air analyzer coupled to the gas extraction accessory for PET bottles. Before starting the experiment, the system was opened, and air was bubbled through the water for 10 minutes to ensure that there was no remaining ^{222}Rn. After that, the system was closed, and ^{222}Rn concentrations were monitored for over 14 h. The increase of ^{222}Rn concentrations in the overlying water of the incubated sediments was linearly approached (errors of 12 – 27%), and the slope was used to calculate the diffusive flux of ^{222}Rn. The diffusive ^{222}Rn flux was 21 ± 8 Bq m^{-2} d^{-1} and accounted for 7.2 – 52% of the total ^{222}Rn inputs. Only BD09 and BD12 had a higher contribution of ^{222}Rn from the sediments to the total ^{222}Rn pool (48 – 97%), likely due to their higher sediment area to water volume ratio compared to deeper lakes.

- *^{222}Rn inputs via inlet streams*

Inputs of ^{222}Rn through the inlet streams (F_{inlet}) were estimated by multiplying the inlet discharge (Q_{inlet}, estimated using a magnetic flow meter or salt slug injections) [m^3 d^{-1}] by the ^{222}Rn concentration in the inlet flow [Bq m^{-3}]. The flux of ^{222}Rn via inlet streams was then estimated by dividing this input by the lake area. Inputs of ^{222}Rn from the inlet streams ranged from 0.46 to 47 Bq m^{-2} d^{-1}. The ^{222}Rn input rate for this source accounted for 0.76 – 27% of the total ^{222}Rn inputs.

- *^{222}Rn inputs via groundwater inflow*

Among all the sources, dissolved ^{226}Ra and diffusion of ^{222}Rn from underlying sediments accounted 7.4 – 55% of the sources in the ^{222}Rn mass balance. Inlet inflows accounted for up to 27% of the total water ^{222}Rn stocks. Combined, these ^{222}Rn sources could not compensate for all ^{222}Rn losses, indicating that groundwater inflow was a major ^{222}Rn source that accounted for 24 – 84% of lake ^{222}Rn sinks.

S.3 ^{222}Rn concentration in groundwater

Groundwater samples (n = 41) were collected from mires at the lake shoreline to determine the ^{222}Rn endmember for the ^{222}Rn mass balance. Additionally, lake bottom sediments (n = 28) were incubated in the lab[7,8] to obtain an independent estimate of ^{222}Rn concentration in groundwater (used for comparison purposes only). The ^{222}Rn concentration from equilibration experiments is the theoretical maximum ^{222}Rn concentration in groundwater entering the system. Briefly, 500 mL PET bottles with Ra-free lake water and about 150-200 g of wet lake sediment were stored for three weeks and periodically shaken. The concentration of ^{222}Rn in the water was measured using the RAD7 coupled to the gas extraction accessory for PET bottles. The ^{222}Rn concentration in groundwater ($C_{Rn,gw}$) was calculated as:

$$C_{Rn,gw} = C_{incubation}\frac{R_{lab}}{R_{field}} \quad \text{(S4)}$$

where $C_{incubation}$ is the measured ^{222}Rn concentration [Bq m^{-3}], and R_{lab} and R_{field} are ratios of volume of water to sediment in the bottle (lab) and in the field (field) (which is a function of the porosity), respectively[9].

There was significant variability in ^{222}Rn concentrations in groundwater samples when considering both field samples (IQR 2100 – 8800 Bq m^{-3}) and sediment incubations (IQR 1800 – 9800 Bq m^{-3}). Large spatial variability in groundwater ^{222}Rn content was also observed at Toolik[10] (range of 10 – 328 Bq m^{-3}) and Landing Lake[11] (range of 15000 – 48000 Bq m^{-3}) in Alaska, likely due to the high lateral and vertical variability of mineral content in peat[12,13]. Despite the high variability, incubation experiments provided similar equilibrium ^{222}Rn concentrations to direct measurements in groundwater (df = 1, F = 0.20, p = 0.65) (Supplementary Figure 9), which suggests that this term is relatively well constrained.

S.4 Uncertainties on groundwater CH_4 inputs to lakes

For those lakes receiving detectable inputs of groundwater, estimations of groundwater inflow rates were heavily dependent on accurate determinations of the ^{222}Rn decay and the atmospheric ^{222}Rn evasion (Supplementary Figure 3). Groundwater inputs of CH_4 are thus highly sensitive to $C_{\mathrm{Rn,lake}}$ and k and, in turn, to their associated uncertainties. By measuring $C_{\mathrm{Rn,lake}}$ in at least 5 different points in each lake (3 for the smallest lake BD12), we likely account for the real spatial variability in $C_{\mathrm{Rn,lake}}$ and obtain a representative $C_{\mathrm{Rn,lake}}$ for each lake. The uncertainty associated with k partially results from the estimation of the gas transfer velocity, thus being highly sensitive to the wind speed. Using the 1h-average wind speed over

the lakes three days prior to sampling and weighting for stronger degassing effects in ^{222}Rn concentrations to concentrations closer to the sampling time, we tried to account for the real variability in the wind speed and better constrain U_{10} [6].

The empirical parametrization of k as a function of wind speed and lake area is also a critical source of uncertainty on the determination of the ^{222}Rn flux to the atmosphere, and thus, on ^{222}Rn-derived estimates of groundwater inflows[2,6]. By using other empirical models to determine k, we would have obtained ^{222}Rn-derived groundwater fluxes from 3% lower (using Cole & Caraco[14]) to 25% higher (using Vachon & Prairie[15]) than the ones reported here. Similarly, CH_4 emissions to the atmosphere would have been overestimated from 4 to 46%. However, the selection of the empirical model to estimate k does not affect the correlation found between groundwater inflows and atmospheric CH_4 fluxes (Figure 3), as k_{Rn}/k_{CH4} ratio did not vary between methods (p = 0.739). We thus consider that the potential uncertainties associated to the parametrization of k do not affect the conclusions of this study.

An additional source of uncertainty is derived from the inherent variability of ^{222}Rn and CH_4 concentrations in groundwater, which may vary up to two orders of magnitude. Since we used the concentrations of ^{222}Rn and CH_4 in all groundwater samples collected for deriving groundwater inflows and CH_4 inputs, the variability associated with these estimates is closely linked with that of the end members.

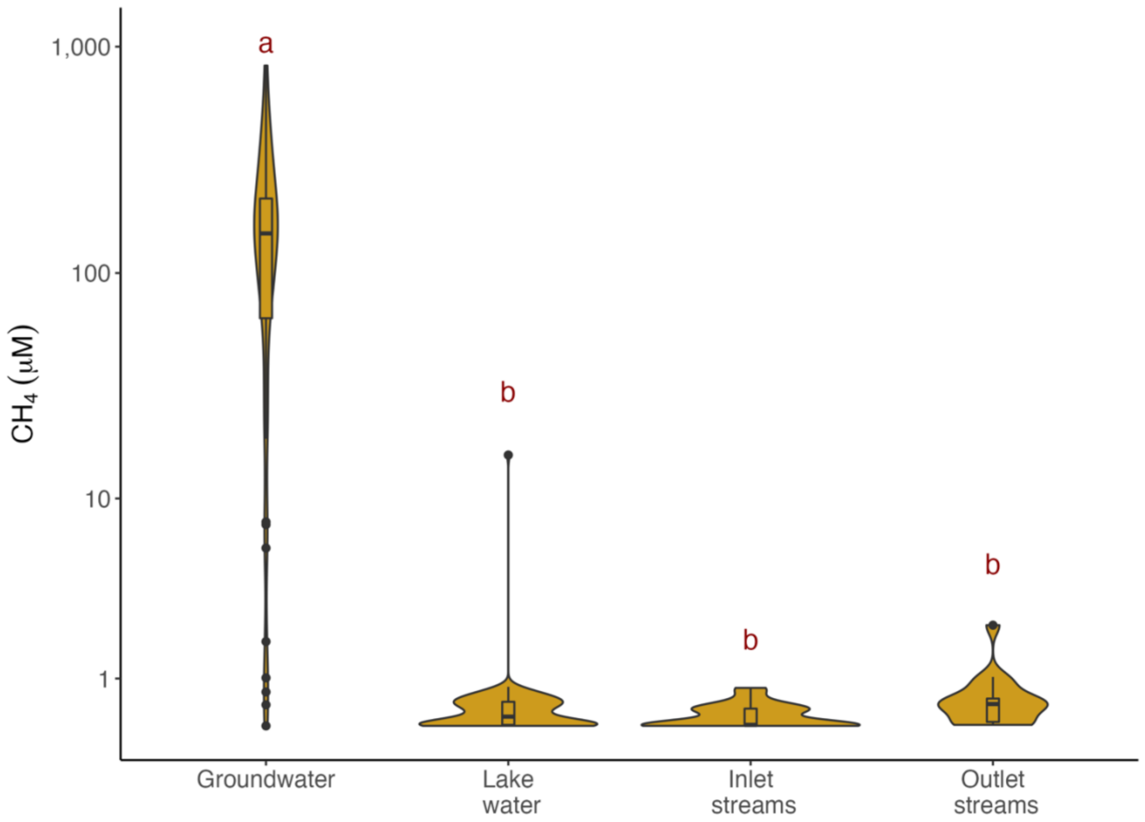

Supplementary Figure 1. **CH_4 concentration in different water sources.** Violin plots include all the samples collected in all sites (*n* = 10) and seasons (*n* = 2) for groundwater (*n* = 41), lake (*n* =191) and inlet (*n*= 31) and outlet (*n* = 22) stream waters. The boundaries of each box plot indicate the interquartile range (25th and 75th percentiles), points indicate outliers, and the solid line in each box marks the median. Different letters above the violin plots indicate statistically significant differences between water sources.

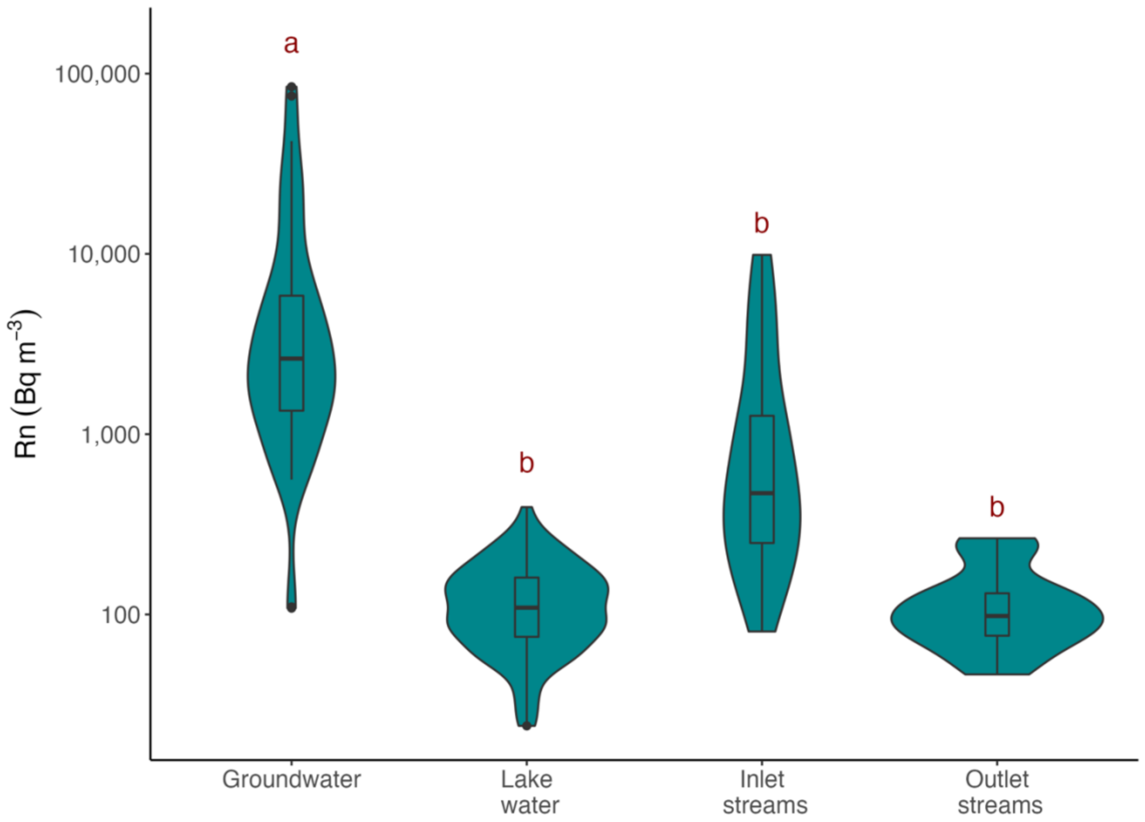

Supplementary Figure 2. **^{222}Rn concentration in different water sources**. Violin plots include all the samples collected in all sites (*n* = 10) and seasons (*n* = 2) for groundwater (*n* = 41), lake (n =191) and inlet (n = 31) and outlet (*n* = 22) stream waters. The boundaries of each box plot indicate the interquartile range (25th and 75th percentiles), points indicate outliers, and the solid line in each box marks the median. Different letters above the violin plots indicate statistically significant differences between water sources.

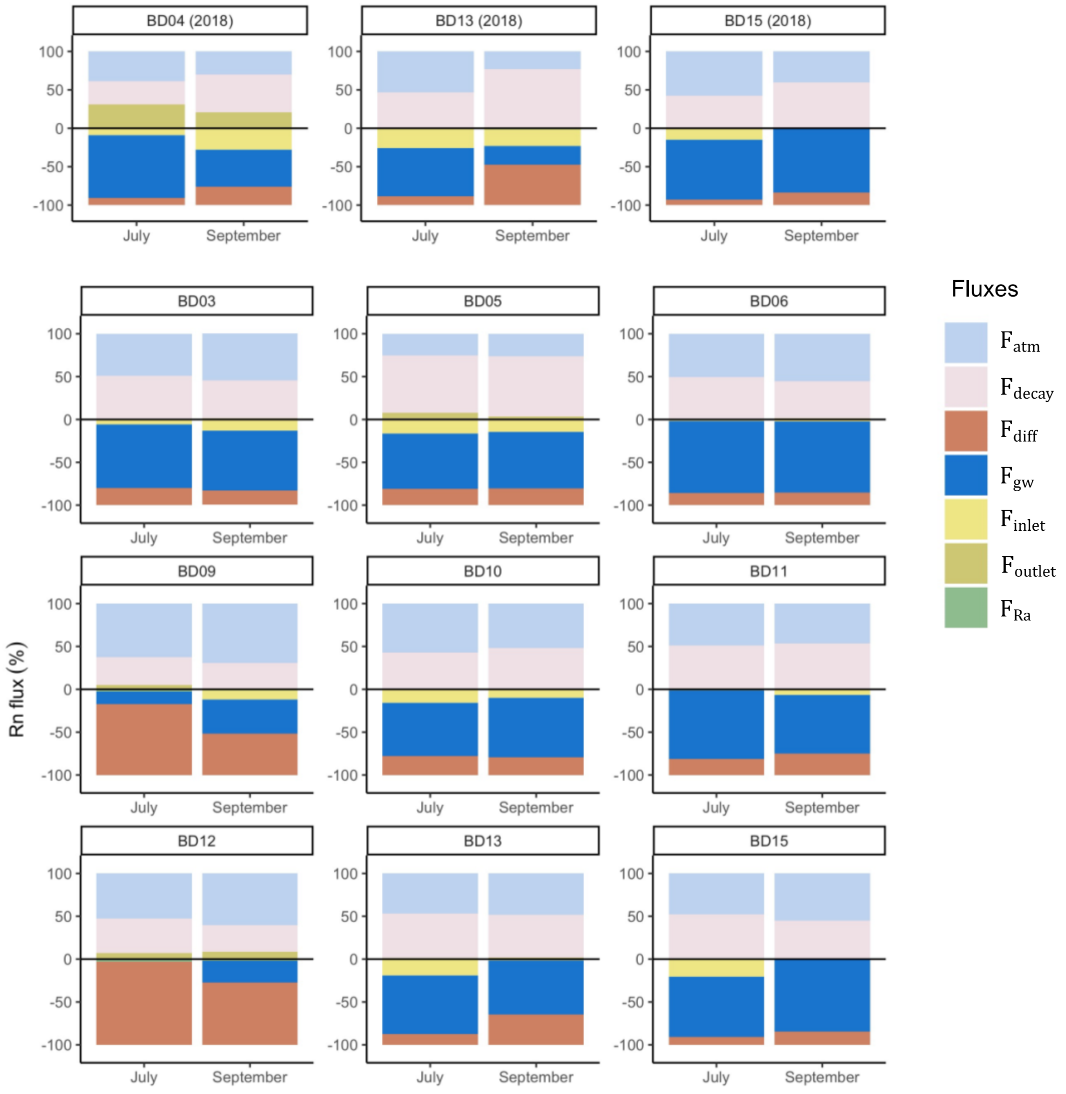

Supplementary Figure 3. **Contribution (in %) of each ^{222}Rn source (negative values) and sink (positive values) in the ^{222}Rn mass balance for the study lakes.** ^{222}Rn sources include discharge from the inlets streams (F_{inlet}), *in situ* ^{222}Rn production from decaying ^{226}Ra dissolved in the water column (F_{Ra}), diffusion of ^{222}Rn from bottom sediments (F_{diff}), and groundwater inflow (F_{gw}). ^{222}Rn sinks include radioactive decay (F_{decay}), evasion to the atmosphere (F_{atm}), and losses through the outlet streams (F_{outlet}).

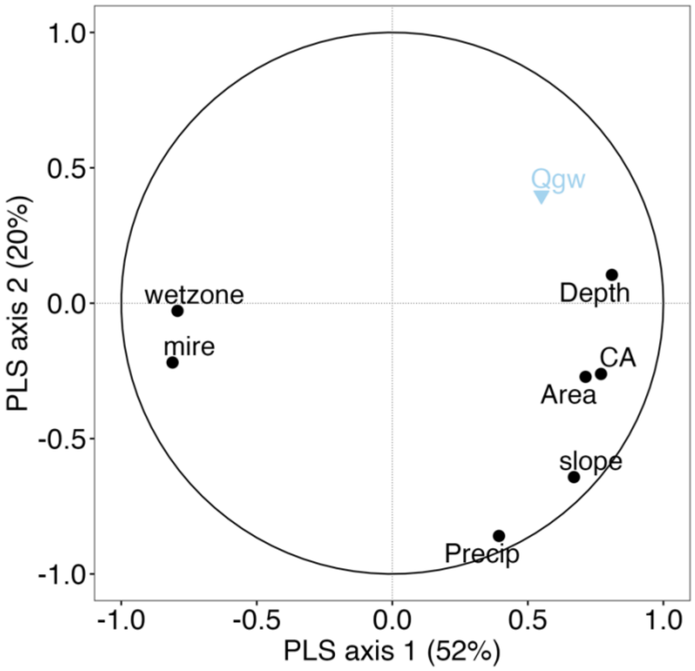

Supplementary Figure 4. **PLS score plot on principal components describing variability with groundwater inflow (Q_{gw}) as response variable and catchment, lake characteristics, and precipitation as predicting variables.** Percentages of the variance explained by the principal component axes are given in parentheses. Area and Depth refer to the mean area and depth of the lake, respectively, CA refers to catchment area, slope refers to the catchment slope, Precip refers to the mean precipitation, and Wetzone and Mire refers to the cover percentage of wet zone and mires in the catchment.

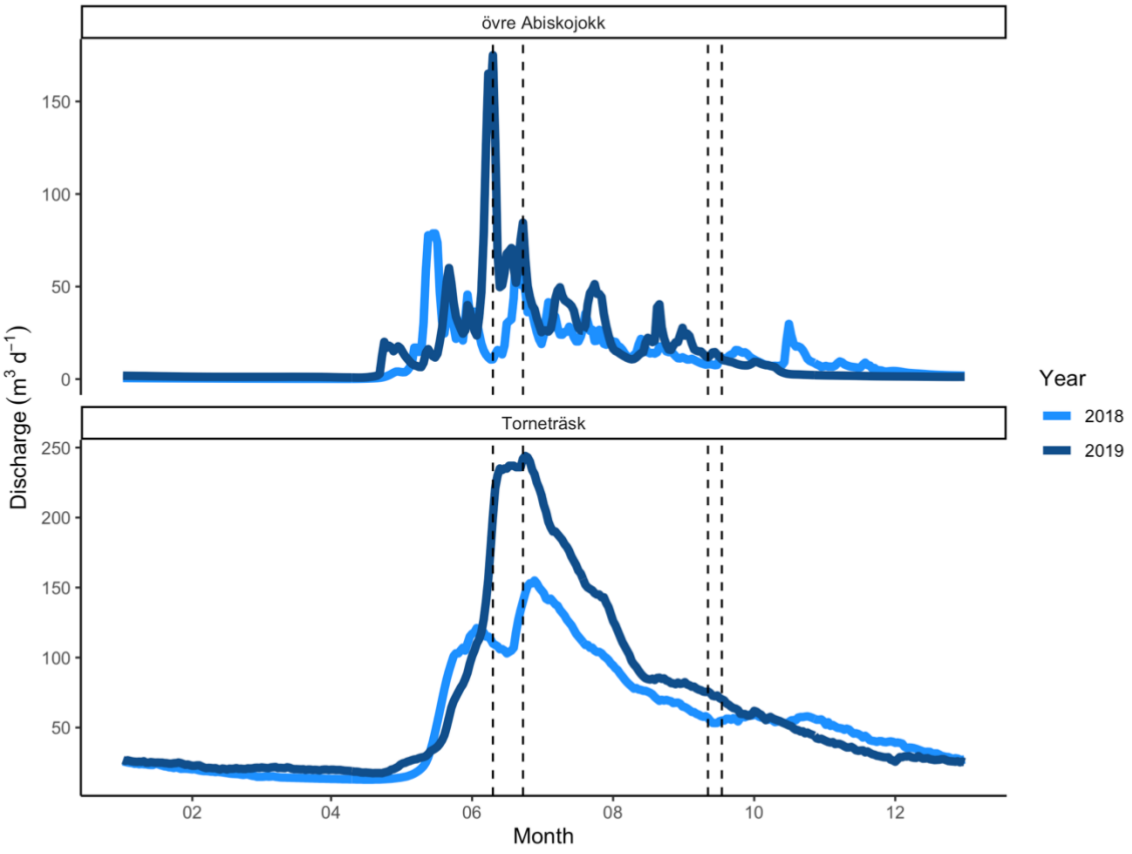

Supplementary Figure 5. **Seasonal discharge patterns in nearby gauging stations.** Discharge in Övre Abiskojokk (draining into Tornetrask) and Torneträsk lake outlet (Data available through the SITES portal https://data.fieldsites.se/portal/). Dashed lines indicate sampling occasions.

173

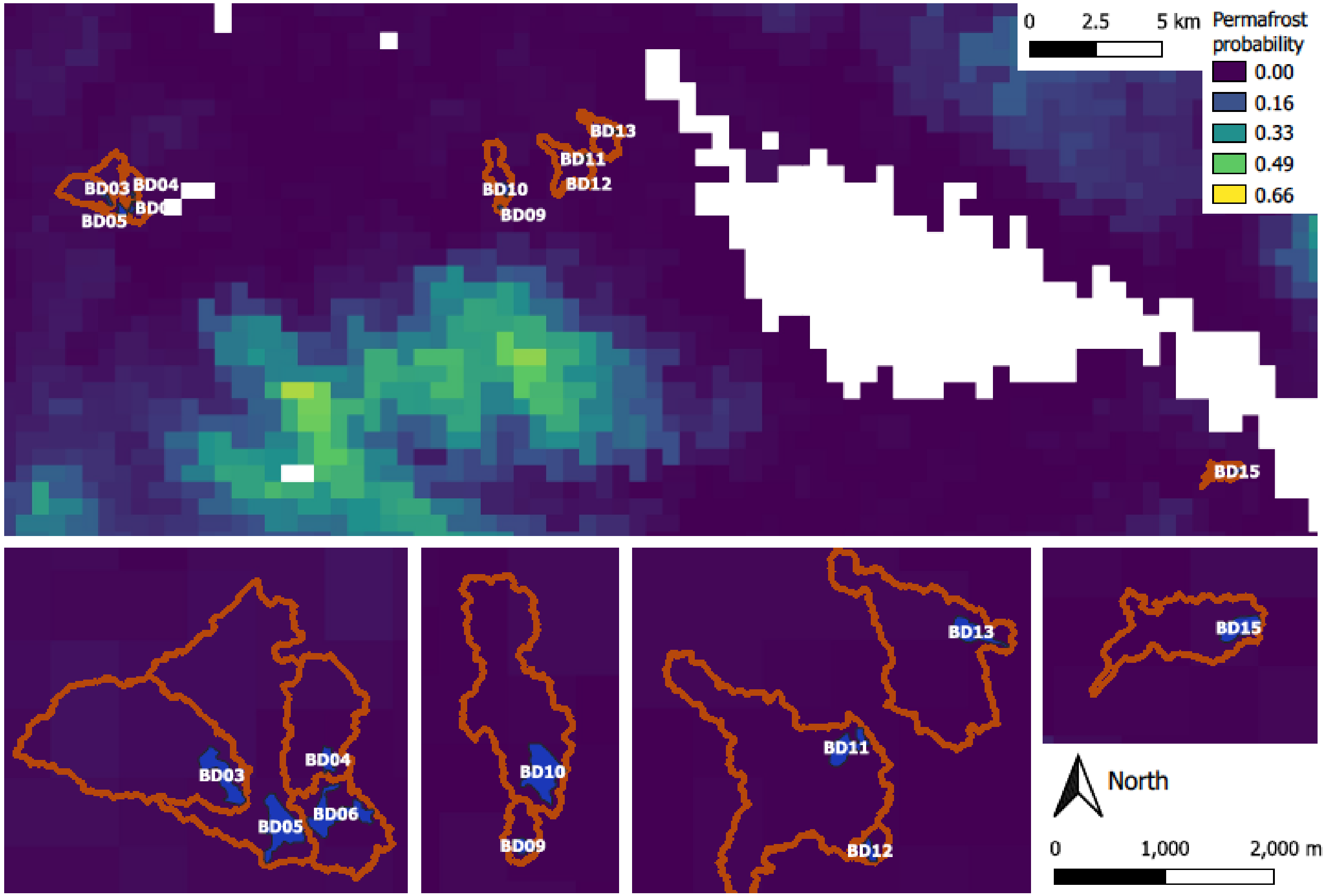

Supplementary Figure 6. **Map of the permafrost distribution of the study area**[16]. Blue color indicates the study lakes and red lines show the corresponding catchments.

174

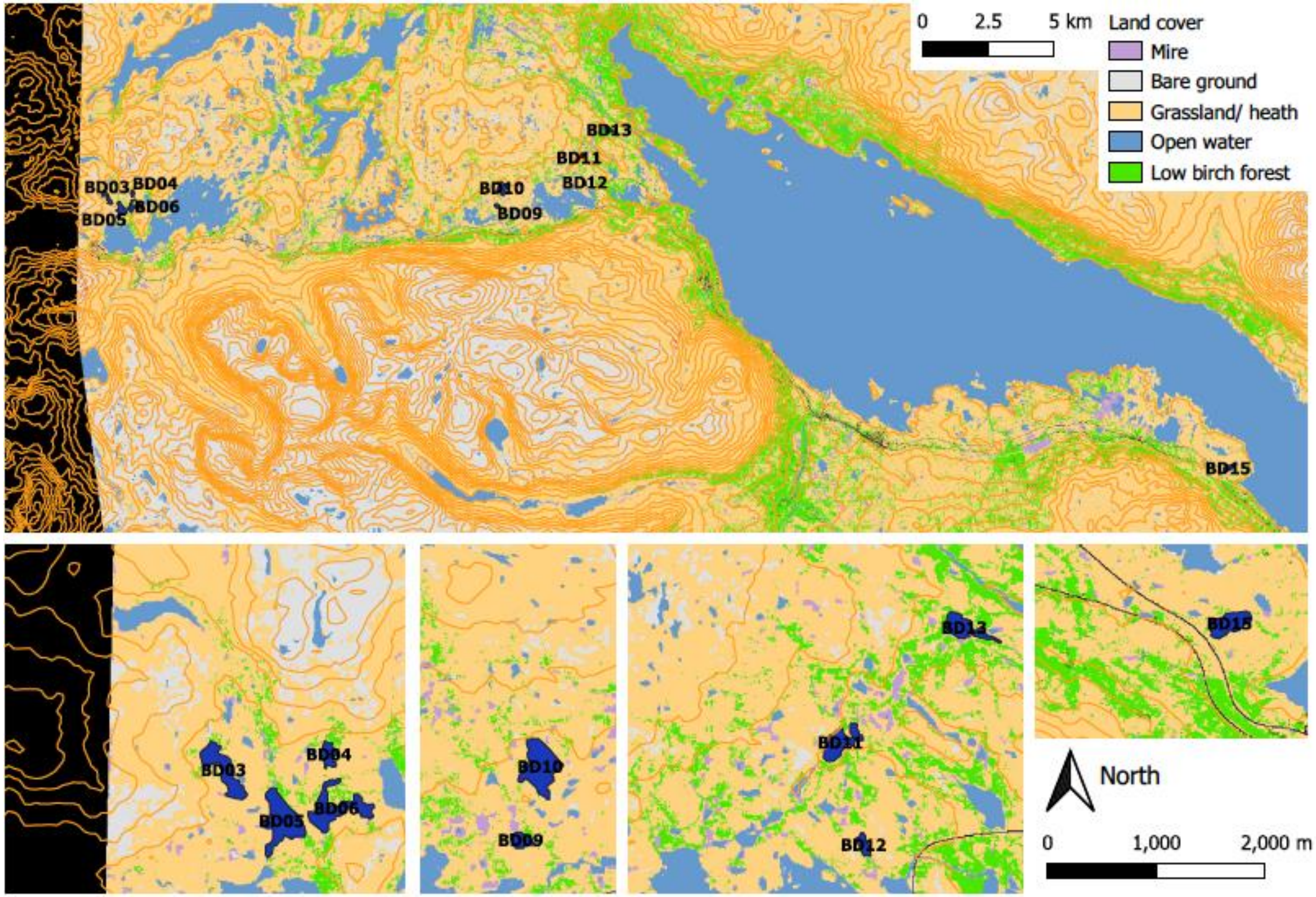

Supplementary Figure 7. **Land cover map of the study area for 2021.** (Image source: ©Lantmäteriet, 2021).

175

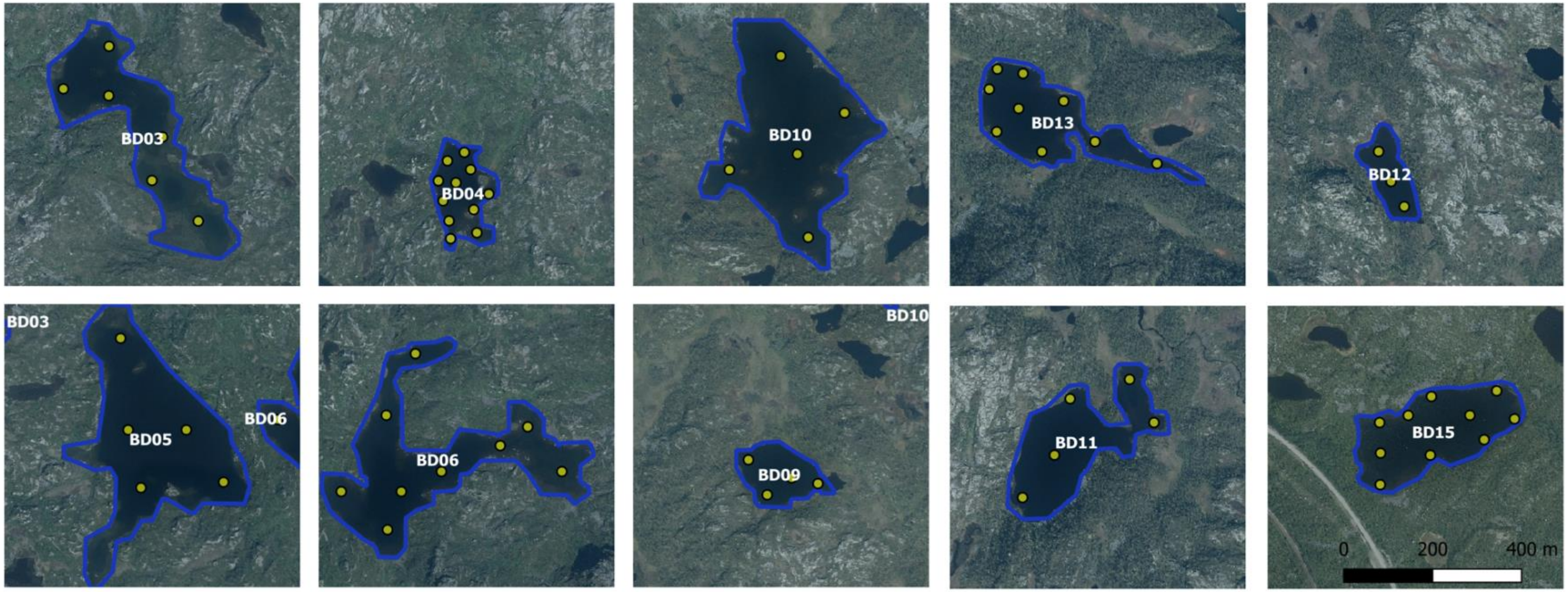

Supplementary Figure 8. **Map of the study lakes and sampling points.** Yellow points indicate locations where water samples were collected. (Image source: ©Lantmäteriet, 2021).

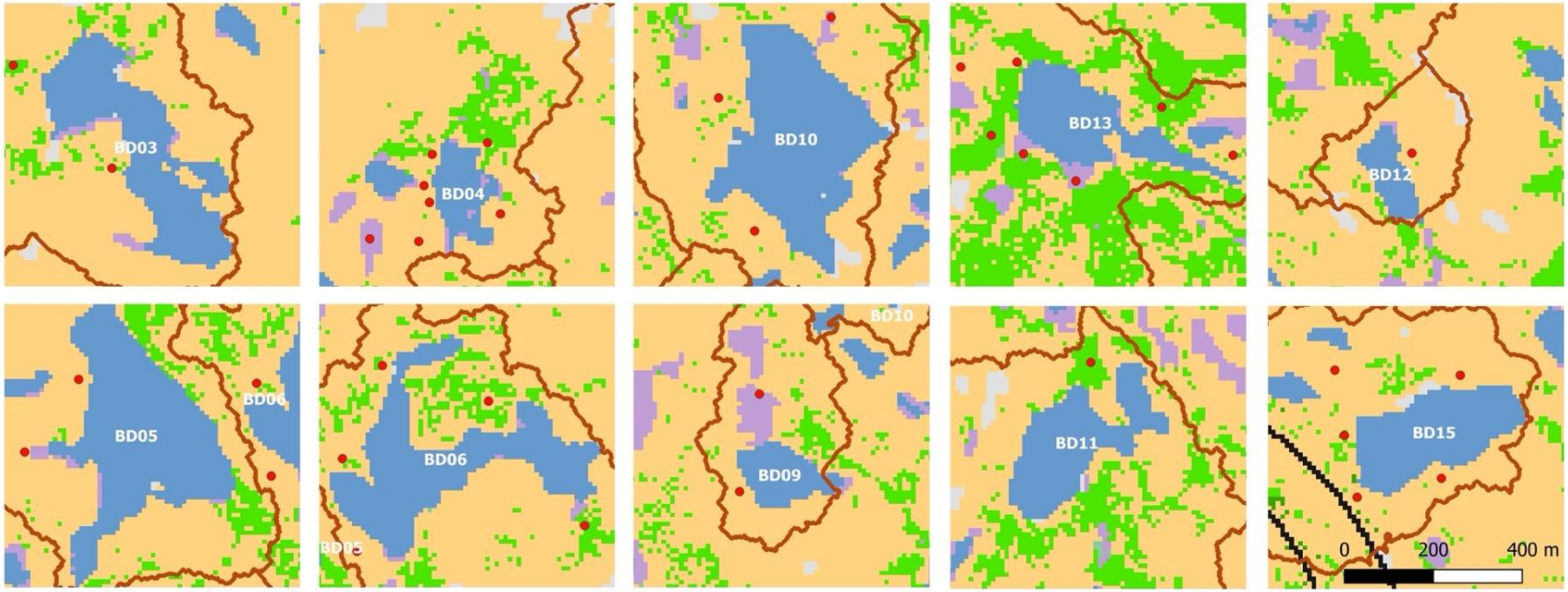

Supplementary Figure 9. **Map of the study lakes and groundwater sampling points.** Red points indicate locations where groundwater samples were collected (Background is landcover map from ©Lantmäteriet, 2021).

178

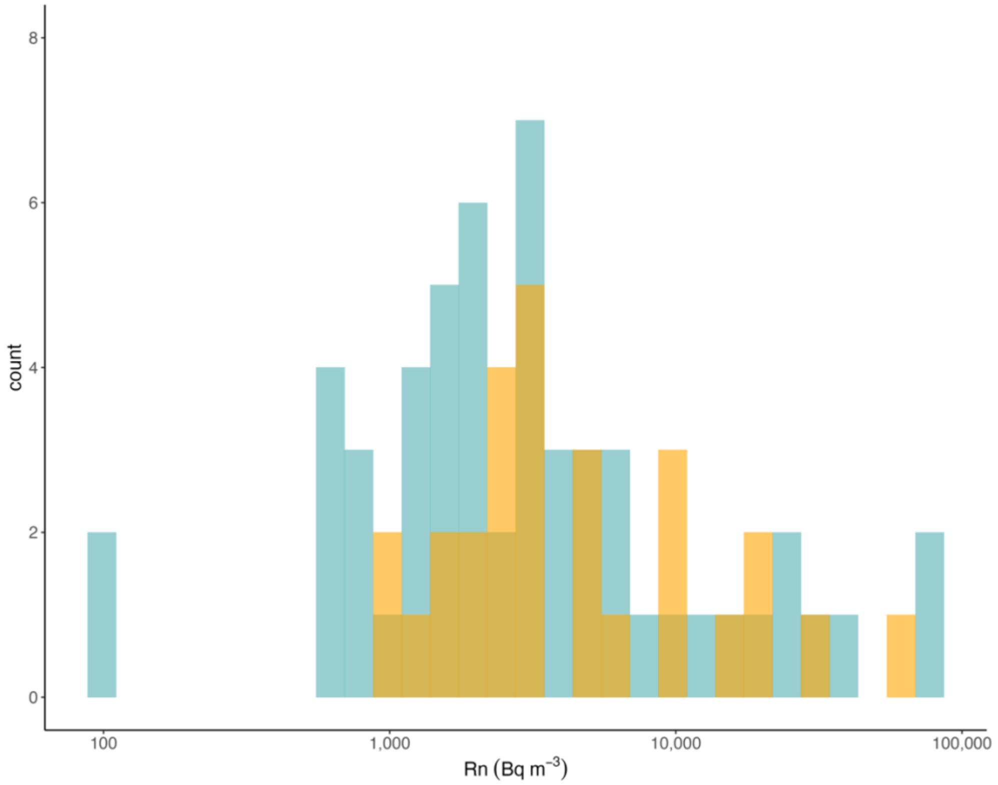

Supplementary Figure 10. **Histogram of ^{222}Rn concentration in groundwater.** ^{222}Rn concentrations were directly measured from groundwater samples (blue) and estimated from sediment incubation experiments (yellow).

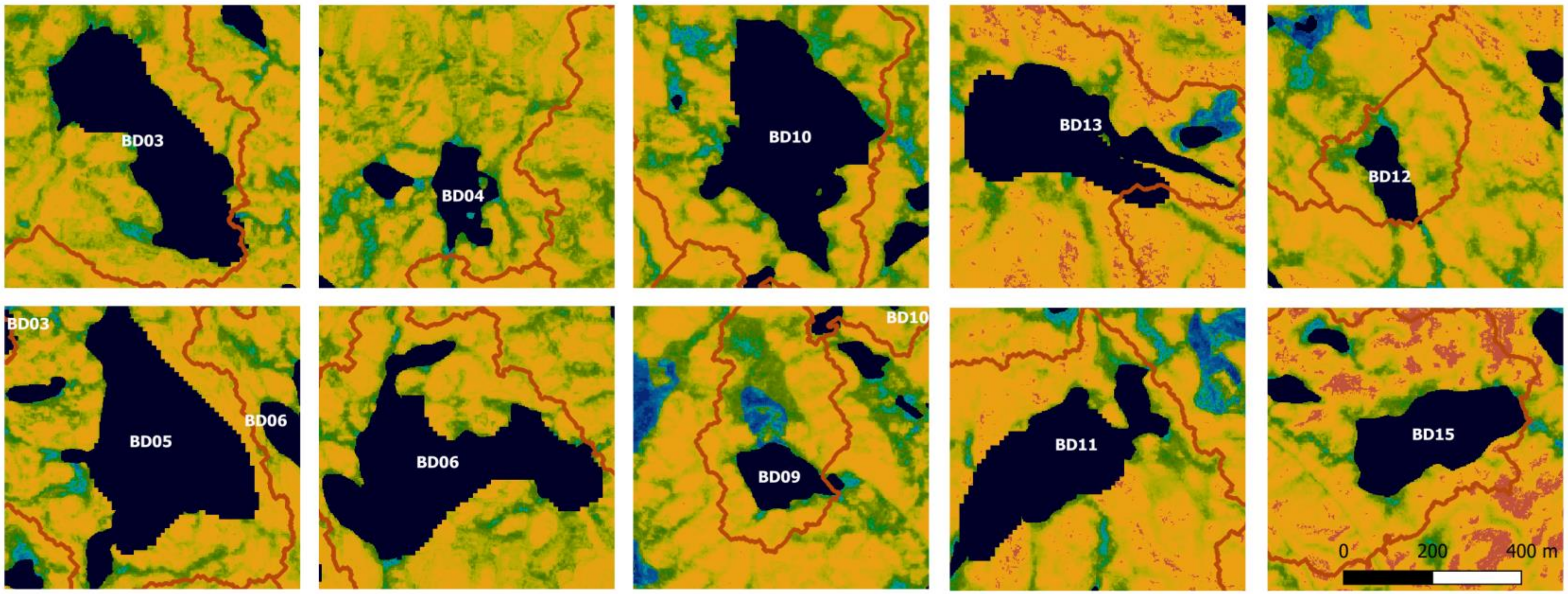

Supplementary Figure 11. **Map of wet areas of the study area for 2021.** (Image source: ©Lantmäteriet, 2021).

Supplementary Table 1. **Definition of the parameters and terms used in the ^{222}Rn mass balance based on Eq.(1) for lakes samples in 2018.** The approach used to estimate each parameter and term, and the corresponding uncertainties are provided in Table S3. Groundwater discharge rates are reported as the median and interquartile range (25th and 75th percentiles).

Term	Definition	Lake						Unit
		BD04		BD13		BD15		
		July	September	July	September	July	September	
Radon balance								
$C_{Rn,gw}$	^{222}Rn concentration in groundwater	1900 - 11000	1900 - 11000	1900 - 11000	1900 - 11000	1900 - 11000	1900 - 11000	Bq m^{-3}
$C_{Ra,lake}$	^{226}Ra concentration in lake water	16.0 ± 7.4	16.0 ± 7.4	16.0 ± 7.4	16.0 ± 7.4	16.0 ± 7.4	16.0 ± 7.4	Bq m^{-3}
$C_{Rn,lake}$	^{222}Rn concentration in lake water	164 ± 13	105.3 ± 7.8	180 ± 18	64.9 ± 5.1	206 ± 19	129 ± 10	Bq m^{-3}
λ	^{222}Rn decay constant	0.181	0.181	0.181	0.181	0.181	0.181	d^{-1}
Rn fluxes								
$\lambda VC_{Ra,lake}$	^{222}Rn production from ^{226}Ra decay	14.8 ± 3.2	14.8 ± 3.2	32.0 ± 7.0	32.0 ± 7.0	38.1 ± 8.3	38.1±8.3	·10^{3} Bq d^{-1}
$F_{diff}A$	^{222}Rn diffusion from sediments	470 ± 190	470 ± 190	1017 ± 400	1017 ± 400	1200 ± 480	1200 ± 480	·10^{3} Bq d^{-1}
$F_{atm}A$	^{222}Rn evasion to the atmosphere	1900 ± 150	590 ± 44	4700± 460	450 ± 35	9700± 890	3030 ± 220	·10^{3} Bq d^{-1}
$\lambda VC_{Rn,lake}$	^{222}Rn loss by decay	1500 ± 120	970 ± 72	4070 ± 400	1500 ± 120	7090 ± 650	44003± 330	·10^{3} Bq d^{-1}
$Q_{inlet}C_{Rn,inlet}$	^{222}Rn input from inlet streams	440 ± 460	530 ± 130	2300 ± 1200	420± 400	2500 ± 1020	dried	·10^{3} Bq d^{-1}
$Q_{outlet}C_{Rn,outlet}$	^{222}Rn output from outlet streams	1600 ± 650	410 ± 330	75 ± 12	21.3 ± 4.1	104 ± 13	64.8 ± 3.3	·10^{3} Bq d^{-1}
$Q_{gw}C_{Rn,gw}$	^{222}Rn flux from groundwater	4100 ± 840	960 ± 410	5600 ± 980	472 ± 579	13000 ± 1600	6300 ± 630	·10^{3} Bq d^{-1}
Q_{gw}	Groundwater discharge	4.9 (1.8 – 8.8)	1.0 (0.35 –2.1)	3.2 (1.1 – 5.6)	0.18 (0.014 – 0.60)	6.4 (2.2 – 11)	3.2 (1.0 – 1.5)	cm d^{-1}

Supplementary Table 2. **Definition of the parameters and terms used in the ^{222}Rn mass balance based on Eq.(1) for lakes samples in 2019.** The approach used to estimate each parameter and term, and the corresponding uncertainties are provided in Table S3. Groundwater discharge rates are reported as the median and interquartile range (25th and 75th percentiles).

Term	Definition	Lake						Unit
		BD03		BD05		BD06		
		July	September	July	September	July	September	
Radon balance								
$C_{Rn,gw}$	^{222}Rn concentration in groundwater	1900 - 11000	1900 - 11000	1900 - 11000	1900 - 11000	1900 - 11000	1900 - 11000	Bq m^{-3}
$C_{Ra,lake}$	^{226}Ra concentration in lake water	16.0 ± 7.4	16.0 ± 7.4	16.0 ± 7.4	16.0 ± 7.4	16.0 ± 7.4	16.0 ± 7.4	Bq m^{-3}
$C_{Rn,lake}$	^{222}Rn concentration in lake water	149 ± 28	154 ± 20	63.2 ± 6.7	64.9 ± 9.0	148 ± 33	126 ± 20	Bq m^{-3}
λ	^{222}Rn decay constant	0.181	0.181	0.181	0.181	0.181	0.181	d^{-1}
Rn fluxes								
$\lambda VC_{Ra,lake}$	^{222}Rn production from ^{226}Ra decay	54 ± 12	54 ± 12	77 ± 17	77 ± 17	65 ± 14	65 ± 14	·10^{3} Bq d^{-1}
$F_{diff}A$	^{222}Rn diffusion from sediments	1700 ± 690	1700 ± 690	2400 ± 970	2400 ± 970	2060 ± 820	2060 ± 820	·10^{3} Bq d^{-1}
$F_{atm}A$	^{222}Rn evasion to the atmosphere	4300 ± 790	5700 ± 750	3300 ± 350	3300 ± 460	7400 ± 1600	7800 ± 1200	·10^{3} Bq d^{-1}
$\lambda VC_{Rn,lake}$	^{222}Rn loss by decay	4400 ± 820	4500 ± 600	8600 ± 910	8800 ± 1200	7100 ± 1600	6090 ± 980	·10^{3} Bq d^{-1}
$Q_{inlet}C_{Rn,inlet}$	^{222}Rn input from inlet streams	450 ± 100	1300 ± 130	2030 ± 310	1700 ± 230	240 ± 34	290 ± 50	·10^{3} Bq d^{-1}
$Q_{outlet}C_{Rn,outlet}$	^{222}Rn output from outlet streams	64 ± 12	100 ± 31	990 ± 230	410 ± 140	141 ± 32	230 ± 45	·10^{3} Bq d^{-1}
$Q_{gw}C_{Rn,gw}$	^{222}Rn flux from groundwater	6500 ± 1300	7200 ± 1200	8300 ± 1400	8300 ± 1700	12000 ± 2400	12000 ± 1800	·10^{3} Bq d^{-1}
Q_{gw}	Groundwater discharge	2.2 (0.73 – 3.8)	2.5 (0.84 – 4.3)	2.1 (0.71 – 3.4)	1.9 (0.68 – 3.5)	3.5 (1.2 – 6.2)	3.4 (1.2 – 5.9)	cm d^{-1}

Supplementary Table 2 (Continued). **Definition of the parameters and terms used in the ^{222}Rn mass balance based on Eq.(1) for lakes samples in 2019.** The approach used to estimate each parameter and term, and the corresponding uncertainties are provided in Table S3. Groundwater discharge rates are reported as the median and interquartile range (25th and 75th percentiles).

		Lake						Unit
Term	Definition	BD09		BD10		BD11		
		July	September	July	September	July	September	
Radon balance								
$C_{Rn,gw}$	^{222}Rn concentration in groundwater	1900 - 11000	1900 - 11000	1900 - 11000	1900 - 11000	1900 - 11000	1900 - 11000	Bq m^{-3}
$C_{Ra,lake}$	^{226}Ra concentration in lake water	16.0 ± 7.4	16.0 ± 7.4	16.0 ± 7.4	16.0 ± 7.4	16.0 ± 7.4	16.0 ± 7.4	Bq m^{-3}
$C_{Rn,lake}$	^{222}Rn concentration in lake water	40.3 ± 5.6	63 ± 31	103 ± 19	122 ± 18	97 ± 19	76.6 ± 7.4	Bq m^{-3}
λ	^{222}Rn decay constant	0.181	0.181	0.181	0.181	0.181	0.181	d^{-1}
Rn fluxes								
$\lambda V C_{Ra,lake}$	^{222}Rn production from ^{226}Ra decay	13.9 ± 3.0	13.9 ± 3.0	70 ± 15	70 ± 15	37.5 ± 8.2	37.5 ± 8.2	·10^3 Bq d^{-1}
$F_{diff} A$	^{222}Rn diffusion from sediments	440 ± 180	440 ± 180	2200 ± 880	2200 ± 880	1200 ± 470	1200 ± 470	·10^3 Bq d^{-1}
$F_{atm} A$	^{222}Rn evasion to the atmosphere	340 ± 46	640 ± 32	5800 ±1050	5500 ± 820	3100 ± 620	2200 ± 210	·10^3 Bq d^{-1}
$\lambda V C_{Rn,lake}$	^{222}Rn loss by decay	173 ± 24	270 ± 135	4300 ± 780	5050 ± 750	3200 ± 640	2500 ± 244	·10^3 Bq d^{-1}
$Q_{inlet} C_{Rn,inlet}$	^{222}Rn input from inlet streams	dried	98 ± 48	1500 ± 160	970 ± 100	58 ± 45	288 ± 35	·10^3 Bq d^{-1}
$Q_{outlet} C_{Rn,outlet}$	^{222}Rn output from outlet streams	27.0 ± 6.5	8.8 ± 2.2	25.3 ± 6.0	71 ± 16	78 ± 16	38 ± 13	·10^3 Bq d^{-1}
$Q_{gw} C_{Rn,gw}$	^{222}Rn flux from groundwater	79 ± 180	360 ± 390	6300 ± 1600	7400 ± 1400	5100 ± 1000	3200 ± 580	·10^3 Bq d^{-1}
Q_{gw}	Groundwater discharge	0.040 (-0.023 – 0.28)	0.31 (0.042 – 0.99)	1.6 (0.55 – 2.7)	1.9 (0.64 – 3.4)	2.4 (0.89 – 4.3)	1.6 (0.57 – 2.9)	cm d^{-1}

Supplementary Table 2 (Continued). **Definition of the parameters and terms used in the ^{222}Rn mass balance based on Eq.(1) for lakes samples in 2019.** The approach used to estimate each parameter and term, and the corresponding uncertainties are provided in Table S3. Groundwater discharge rates are reported as the median and interquartile range (25th and 75th percentiles).

Term	Definition	Lake						Unit
		BD12		BD13		BD15		
		July	September	July	September	July	September	
Radon balance								
$C_{Rn,gw}$	^{222}Rn concentration in groundwater	1900 - 11000	1900 - 11000	1900 - 11000	1900 - 11000	1900 - 11000	1900 - 11000	Bq m^{-3}
$C_{Ra,lake}$	Ra concentration in lake water	16.0 ± 7.4	16.0 ± 7.4	16.0 ± 7.4	16.0 ± 7.4	16.0 ± 7.4	16.0 ± 7.4	Bq m^{-3}
$C_{Rn,lake}$	^{222}Rn concentration in lake water	28.9 ± 3.2	36.8± 6.4	190 ± 64	63.6 ± 6.9	198 ± 11	103.1 ± 6.9	Bq m^{-3}
λ	^{222}Rn decay constant	0.181	0.181	0.181	0.181	0.181	0.181	d^{-1}
Rn fluxes								
$\lambda V C_{Ra,lake}$	^{222}Rn production from ^{226}Ra decay	12.1± 2.6	12.1± 2.6	3.2 ± 7.0	3.2 ± 7.0	38.1± 8.3	38.1± 8.3	$\cdot 10^3$ Bq d^{-1}
$F_{diff} A$	^{222}Rn diffusion from sediments	390 ± 150	390 ± 150	1020 ± 400	1020 ± 400	1200 ± 480	1200 ± 480	$\cdot 10^3$ Bq d^{-1}
$F_{atm} A$	^{222}Rn evasion to the atmosphere	170 ± 19	320 ± 55	3900 ± 580	1400 ± 150	6300 ± 340	4400 ± 300	$\cdot 10^3$ Bq d^{-1}
$\lambda V C_{Rn,lake}$	^{222}Rn loss by decay	130 ± 14	164 ± 28	4300 ± 650	1400 ± 160	6800 ± 370	3500 ± 240	$\cdot 10^3$ Bq d^{-1}
$Q_{inlet} C_{Rn,inlet}$	^{222}Rn input from inlet streams	no inlet	no inlet	1500 ± 200	21.9 ± 3.6	2600 ± 270	dried	$\cdot 10^3$ Bq d^{-1}
$Q_{outlet} C_{Rn,outlet}$	^{222}Rn output from outlet streams	22.6 ± 2.6	46 ± 32	87 ± 11	54.3 ± 2.8	69 ± 10	15.0 ± 2.2	$\cdot 10^3$ Bq d^{-1}
$Q_{gw} C_{Rn,gw}$	^{222}Rn flux from groundwater	-78 ± 160	130 ± 170	5600 ± 980	1800 ± 460	9300 ± 750	6700 ± 610	$\cdot 10^3$ Bq d^{-}
Q_{gw}	Groundwater discharge	-0.047 (-0.28 – 0.023)	0.11 (0.0087 –0.38)	3.2 (1.1 – 5.7)	0.99 (0.37 – 1.7)	4.6 (1.5 – 8.0)	3.4 (1.1 – 5.7)	cm d^{-1}

Supplementary Table 3. **Parameters and terms of the ^{222}Rn mass balance and the approach used for their uncertainties.**

Parameter	Unit	Definition	Estimation of parameter	Estimation of uncertainty
V	[m^3]	Lake volume	Interpolation based on bathymetry using ReefMaster software	Assumed 5%
A	[m^2]	Lake area	Interpolation based on bathymetry using ReefMaster software	Assumed 5%
$C_{Rn,gw}$	[Bq m^{-3}]	^{222}Rn concentration in groundwater	1000 Monte Carlo simulations	Interquartile range of 1000 Monte Carlo simulations
$C_{Ra,lake}$	[Bq m^{-3}]	^{226}Ra concentration in lake waters	Average ^{226}Ra concentration in lake water	Analytical uncertainty and standard deviation
$C_{Rn,lake}$	[Bq m^{-3}]	^{222}Rn concentration in lake waters	Average ^{222}Rn concentration in lake water	Analytical uncertainty and standard deviation
$C_{Rn,inlet}$	[Bq m^{-3}]	^{222}Rn concentration in inlet streams	^{222}Rn concentration in inlet water	Analytical uncertainty
$C_{Rn,outlet}$	[Bq m^{-3}]	^{222}Rn concentration in outlet streams	^{222}Rn concentration in outlet water	Analytical uncertainty
Q_{inlet}	[m^3 d^{-1}]	Inlet stream discharge	Salt slug injections or flow meter measurements	Assumed 10%
Q_{outlet}	[m^3 d^{-1}]	Outlet stream discharge	Salt slug injections or flow meter measurements	Assumed 10%
λ	[d^{-1}]	^{222}Rn decay constant	Known	-
k_{600}	[m d^{-1}]	Gas transfer velocity	After Klaus and Vachon [2]	Range for all lakes
**Term**		**Definition**	**Estimation of term**	**Estimation of uncertainty**
$\lambda\ V\ C_{Ra,lake}$	[Bq d^{-1}]	^{222}Rn production by ^{226}Ra decay	$\lambda\ V\ C_{Ra,lake}$	Propagated uncertainty
$F_{diff}\ A$	[Bq d^{-1}]	^{222}Rn diffusion from bottom sediments	Diffusion experiments	Propagated measurement error
$F_{atm}\ A$	[Bq d^{-1}]	^{222}Rn evasion to the atmosphere	$k\ (C_{gas,lake} - C_{gas,air})$	Propagated uncertainty
$\lambda\ V\ C_{Rn,lake}$	[Bq d^{-1}]	^{222}Rn decay	$\lambda\ V\ C_{Rn,lake}$	Propagated uncertainty
$Q_{inlet}\ C_{Rn,inlet}$	[Bq d^{-1}]	^{222}Rn inputs from inlet streams	$Q_{inlet}\ C_{Rn,inlet}$	Propagated uncertainty
$Q_{outlet}\ C_{Rn,outlet}$	[Bq d^{-1}]	^{222}Rn outputs through outlet streams	$Q_{outlet}\ C_{Rn,outlet}$	Propagated uncertainty
$Q_{gw}\ C_{Rn,gw}$	[Bq d^{-1}]	Groundwater ^{222}Rn flux	Eq. (1)	Propagated uncertainty

Supplementary References

1. Wanninkhof, R. Relationship between wind speed and gas exchange over the ocean revisited. *Limnol. Oceanogr. Methods* **12**, 351–362 (2014).
2. Klaus, M. & Vachon, D. Challenges of predicting transfer velocity from wind measurements over global lakes. *Aquat. Sci.* **82**, 53 (2020).
3. Crusius, J. & Wanninkhof, R. Gas transfer velocities measured at low wind speed over a lake. *Limnol Ocean.* **48**, 1010–1017 (2003).
4. Dimova, N. T. & Burnett, W. C. Evaluation of groundwater discharge into small lakes based on the temporal distribution of radon-222. *Limnol. Oceanogr.* **56**, 486–494 (2011).
5. Dimova, N. T., Burnett, W. C., Chanton, J. P. & Corbett, J. E. Application of radon-222 to investigate groundwater discharge into small shallow lakes. *J. Hydrol.* **486**, 112–122 (2013).
6. Rodellas, V. *et al.* Conceptual uncertainties in groundwater and pore-water fluxes estimated by radon and radium mass balances. *Limnol. Oceanogr.* 1–19 (2021) doi:10.1002.lno.11678.
7. Chanyotha, S., Kranrod, C. & Burnett, W. C. Assessing diffusive fluxes and pore water radon activities via a single automated experiment. *J. Radioanal. Nucl. Chem.* **301**, 581–588 (2014).
8. Corbett, D. R., Burnett, W. C., Cable, P. H. & Clark, S. B. A multiple approach to the determination of radon fluxes from sediments. *J. Radioanal. Nucl. Chem.* **236**, 247–253 (1998).
9. Stieglitz, T. C., Beek, P., Souhaut, M. & Cook, P. G. Karstic groundwater discharge and seawater recirculation through sediments in shallow coastal Mediterranean laggons, determined from water, salt and radon budgets. *Mar. Chem.* **156**, 73–84 (2013).
10. Paytan, A. *et al.* Methane transport from the active layer to lakes in the Arctic using Toolik Lake, Alaska, as a case study. *Proc. Natl. Acad. Sci.* **112**, 201417392 (2015).
11. Dabrowski, J. S. *et al.* Using radon to quantify groundwater discharge and methane fluxes to a shallow, tundra lake on the Yukon-Kuskokwim Delta, Alaska. *Biogeochemistry* **148**, 69–89

(2020).

12. Morison, M. Q., Macrae, M. L., Petrone, R. M. & Fishback, L. Seasonal dynamics in shallow freshwater pond-peatland hydrovhemical interactions in a subarctic permafrost environment. *Hydrol. Process.* **15**, 462–475 (2017).
13. Pawlowski, D., Okupny, D., Wlodarski, W. & Zielinski, T. Spatial variability of selected physicochemical parameters within peat deposits in small valley mire: a geostatistical approach. *Geologos* **20**, (2014).
14. Cole, J. & Caraco, N. Atmospheric exchange of carbon dioxide in a low-wind oligotrophic lake measured by. *Limnol Ocean.* **43**, (1998).
15. Vachon, D. & Prairie, Y. T. The ecosystem size and shape dependence of gas transfer velocity versus wind speed relationships in lakes. *Can. J. Fish. Aquat. Sci.* **70**, 1–8 (2013).
16. Obu, J. *et al.* Northern Hemisphere permafrost map based on TTOP modelling for 2000-2016 at 1 km2 scale. *Earth-Science Rev.* **193**, 299–316 (2019).
