# Supplementary material for: Groundwater discharge as a driver of methane emissions from Arctic lakes
Source: Nat Commun. 2022 Jun 27;13:3667. doi: 10.1038/s41467-022-31219-1 (PMC9237097; doi:10.1038/s41467-022-31219-1)
Supplement: Supplementary file 3 — Description of Additional Supplementary Files [file 41467_2022_31219_MOESM3_ESM.pdf]

Description of Additional Supplementary Files

File Name: Supplementary Data 1
Description: Compiled values and original sources of CH_4 fluxes in Arctic lakes.

File Name: Supplementary Data 2
Original data used in this study.
